# Supplementary figures and images for: Sampling is decisive to determination of Leishmania (Viannia) species
Source: PLoS Negl Trop Dis. 2024 Apr 25;18(4):e0012113. doi: 10.1371/journal.pntd.0012113 (PMC11045131; doi:10.1371/journal.pntd.0012113)

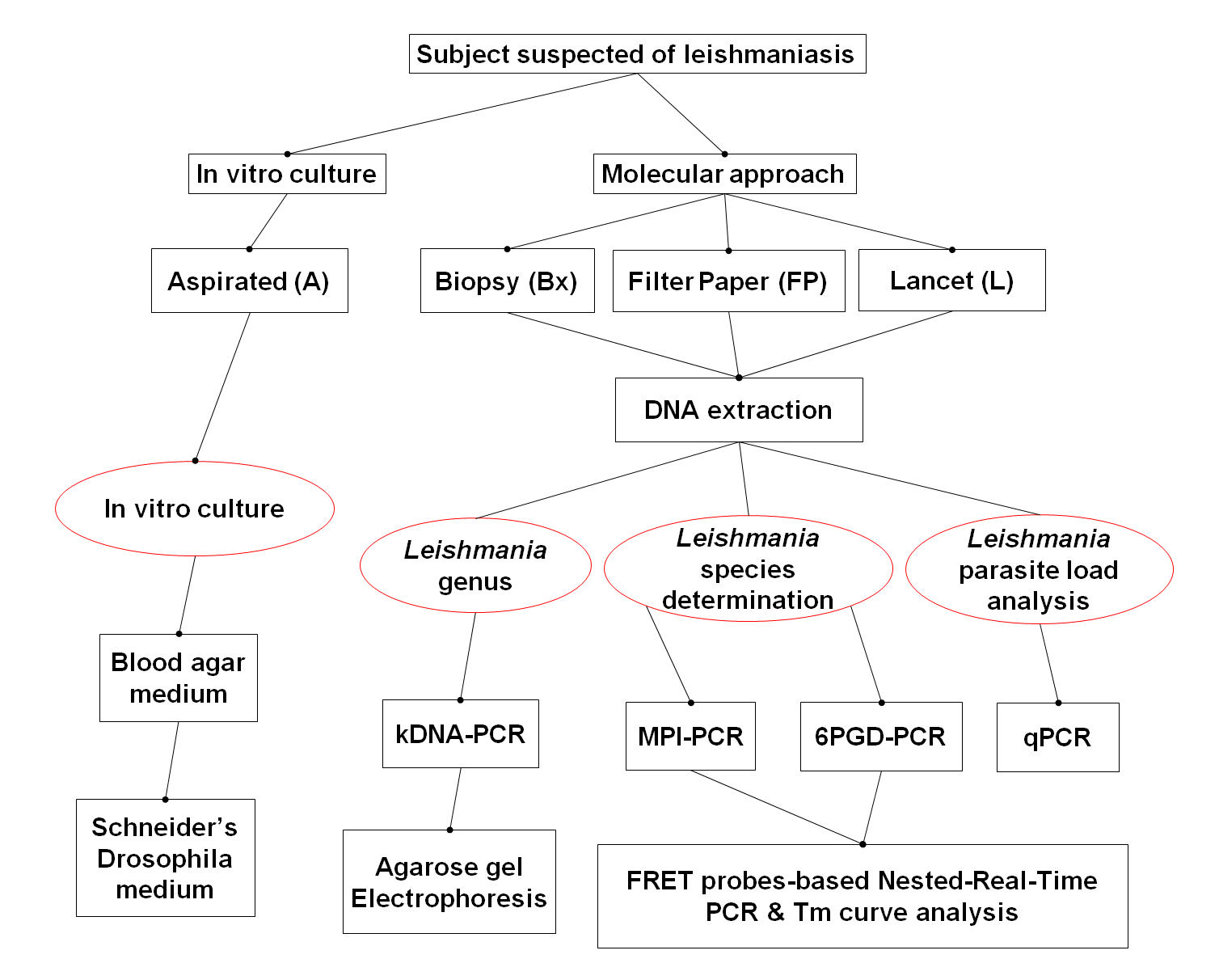

Supplement: S1 Fig — (TIF) [file pntd.0012113.s001.tif]
