# Supplementary material for: Sampling is decisive to determination of Leishmania (Viannia) species
Source: PLoS Negl Trop Dis. 2024 Apr 25;18(4):e0012113. doi: 10.1371/journal.pntd.0012113 (PMC11045131; doi:10.1371/journal.pntd.0012113)
Supplement: Attachment — Submitted filename: Cover and responsesFeb2024.docx [file pntd.0012113.s003.docx]

February 2^nd^, 2024

(PNTD-D-23-01361) - [EMID:b2c56bb03b10b66c] manuscript "Sampling is decisive to determination of *Leishmania* species" for consideration at PLOS Neglected Tropical Diseases

Dear PLOS NTD Editors and Reviewers

We have made appropriate modifications to the manuscript and are confident that we have answered all of the reviewers' questions.

All authors appreciate comments and suggestions that allow us to explain in more detail certain aspects that seemed confusing to the reviewers.

Below we mention the responses and some modifications to the text and certain figures.

Best regards

Maxy B. De los Santos D.

Principal and corresponding author

//

**Methods**

**Reviewer #1:** There is no hypothesis clearly stated, however the aims of the study are clearly articulated.

The design, population studied, and sample size are clearly described and appropriate; the statistical methods seem appropriate and there are no ethical concerns as consent was signed by the patients.

It is unclear though whether the study was blinded at any stage. This should be indicated, and the limitations of a non-blind study discussed.

**Response:** Thank you for the insight. We confirmed that people involved in the different lab procedures were blinded to the other results. The results of culture, kDNA-PCR, and Nested Real-Time PCRs were compiled at the end for the analysis. We hypothesize that different types of samples can produce variations in the results and this variation can be caused due to differences in parasite load.

**Reviewer #2:** See general comments

**Results**

**Reviewer #1:** The analysis and results are well described and supported.

The graph in figure 3B is difficult to read. Please make sure to increase the font and submit a high-quality image.

I also suggest creating a graphical abstract figure or a figure summarizing the various methods used and the main results yielded.

**Response:** The figure 3b was modified on page 17. Regarding to the figure, we designed a flowchart as supplementary material S1 Fig.

**Reviewer #2:** (No Response)

**Conclusions**

**Reviewer #1:** The conclusions are supported and some limitations are mentioned.

The public relevance of the study is addressed.

**Reviewer #2:** (No Response)

**Editorial and Data Presentation Modifications?**

**Reviewer #1:** Minor revisions:

**1)** It is unclear though whether the study was blinded at any stage. This should be indicated, and the limitations of a non-blind study discussed. (See methods part)

**Response:** It was added on page 7, lines 131-134: The different samples obtained were coded and processed blindly, without knowledge of previous or by-pair results from their arrival at the laboratory, to avoid analytical biases during all procedures and stages of this study.

**2)** The graph in figure 3B is difficult to read. Please make sure to increase the font and submit a high-quality image.(See results part)

**Response:** The font was increased, and a high-quality image was submitted for Figure 3B on page 17.

**3)** I also suggest creating a graphical abstract figure or a figure summarizing the various methods used and the main results yielded. (See results part)

**Response:** The flowchart was added as supplementary material S1 Fig. We have mentioned this graph on page 11, line 215 and page 37 line 827. Additional information is in Table 2 and Fig 5.

**4)** What is the prevalence in the territory of the various species of *Leishmania* identified in this article? This needs to be discussed in the introduction or discussion sections.

**Response:** The prevalence of each *Leishmania* species is beyond the scope of this manuscript. It is important to highlight that there is very limited information on *Leishmania* species prevalence and distribution in Peru. However, *Leishmania* species prevalence from our results is shown on page 14 lines 290-296. Also, we discuss on page 22, lines 417 to 423 that *L. (V.) braziliensis* was the most prevalent species according to previous studies, followed by *L. (V.) guyanensis* and *L. (V.) lainsoni*.

**5)** The authors talk about false negatives in the discussion. Is this also addressed in the data? This concept should be made more clear.

**Response:** The different tests have allowed the correct classification of the subjects, even when we have observed a false negative result in some samples. Individuals were considered a case if any of the assays were positive, this was described in methods, page 11, lines 219-224. Additionally, this issue was discussed on page 24, lines 478-487.

**6)** The authors should discuss why it is so important to determine the exact species of Leishmania affecting a certain individual (because of treatment choice, epidemiological research...etc?)

**Response:** The importance of species identification has been shown in multiple articles derived from clinical and surveillance studies. We are mentioning some of the reasons that justify species identification on the pages 24, lines 484-491.

**Reviewer #2: (**No Response)

**Summary and General Comments**

**Reviewer #1:** This is a very interesting manuscript investigating different methods of *Leishmania* detection in order to improve species identification in the field. The results found will have a noticeable public health impact in the territory and could be extrapolated to other endemic areas as well. Overall, there are no major issues and I recommend publication with minor revisions. See my comments above.

**Reviewer #2:** In the paper described by de los santos et al, entitled “sampling is decisive to determination of *Leishmania* species, they compared the sensitivity of molecular identification of *Leishmania* species from Peru, using 3 types of sampling: punch biopsy, filter paper and lancet scraping. They showed that different sampling methods can affect the discrimination capacity of molecular tests for the identification of *Leishmania* species. They demonstrated that lancet scraping is more efficient than filter paper imprints or biopsies to identify more leishmanisais cases. They suggested that scraping with lancets could be used to identify parasite as an alternative to invasive methods such biopsy. It is very interesting study that deals with a topical problem, diagnosis of leishmaniasis, in fact the identification of the parasite are necessary for the efficiency of treatment. However, the authors need to improve the manuscript.

**Major points**

**1.** The authors have to add controls: negative control samples from healthy individuals. They have to check the integrity of extracted DNA by using PCR targeting host gene like **beta globin**. It is very important to check the true negativity. It is also important to compare the recovery of DNA from each sampling methods, especially in the sensitive area (Eyes, lips…).

**Response:** We used samples from healthy individuals as a negative control. The integrity of extracted DNA was determined by electrophoresis using endogenous retrovirus 3 (ERV3) as a marker for human DNA (data not shown) that we used in qPCR assays. Also, the quality or integrity (purity) of the DNA was determined by quantification in a NanoDrop UV Visible Spectrophotometer using the ratio A260/A280. It is important to notice the extracted DNA is a mixture of parasite DNA (nuclear, kinetoplastid, and mitochondrial DNAs) with a variable quantity of human DNA.

We appreciate the suggestion of the inclusion of samples from sensitive areas. However, it is important to consider the epidemiological context of leishmaniasis in Peru. The vast majority of cutaneous infections are located in the limbs, trunk, and back (S1 Table). It would be almost impossible to obtain a reasonable number of samples from cutaneous lesions from sensitive areas for such a comparison.

**2.** It would be interesting to consider the amplification and sequencing of another molecular marker for the identification of *Leishmania* species especially for those that were not identified.

**Response:** Although this topic is outside the scope of our manuscript, we have considered the use of other markers to overcome the drawbacks observed in our study, such as malate dehydrogenase (MDH) and glucose -6-phosphate isomerase (GPI) (published by Tsukayama et al. 2009) and unpublished data using other markers for MLST such as **G6PGD, ICD, ALAT, PGM, ENOL, HGPRT, PMM** (primers published by Boité et al 2012, Marco et al 2015, Herrera et al 2017). Unfortunately, in all cases, the main problem that affects the amplification and sensitivity of the results is the type of sample linked to the parasite load. As can be seen in the six figures below, the presence of weak bands (lanes with red numbers) is very consistent in all markers for the same samples. We also observed that the low or no amplification from the same sample (lanes 2 and 14) probably resulted from parasite load that likely affected the results in some of the markers tested.

On the other hand, NGS and WGA assays could be elicited to overcome the problems observed in the PCRs, but these assays require a high concentration and purity of DNA, but not all samples have it. The main studies use DNA from in vitro cultures of *Leishmania* (promastigotes) to avoid variable amounts of human DNA in the sample and problems during the amplification and sequencing of the parasite's genomic DNA. Domagalska et al 2020 theoretically described the challenges and alternatives to overcome these problems through the enrichment of samples.

**G6PGD, 881 bp, with 26 samples, run 25 May 2022**

**M (-)	(+)	1	2	3	4	5	6	7	8	9	10 11 12 13 14 15 16 17 18 19 20 21 22 23 24 25	26 M**

**ICD, 1022 bp, with 26 samples, run 25 May 2022**

**M (-)	(+)	1	2	3	4	5	6	7	8	9	10 11 12 13 14 15 16 17 18 19 20 21 22 23 24 25	26 M**

**ALAT, 589 bp, with 26 samples, run 26 May 2022**

**M (-)	(+)	1	2	3	4	5	6	7	8	9	10 11 12 13 14 15 16 17 18 19 20 21 22 23 24 25	26 M**

**PGM, 529 bp, with 26 samples, run 26 May 2022**

**M (-)	(+)	1	2	3	4	5	6	7	8	9	10 11 12 13 14 15 16 17 18 19 20 21 22 23 24 25	26 M**

**ENOL, 431 bp, with 26 samples, run 26 May 2022**

**M (-)	(+)	1	2	3	4	5	6	7	8	9	10 11 12 13 14 15 16 17 18 19 20 21 22 23 24 25	26 M**

**PMM, 536 bp, with 26 samples, run 27 May 2022**

**M	(-)	(+) 1	2	3	4	5	6	7	8	9	10 11 12 13 14 15 16 17 18 19 20 21 22 23 24 25	26 M**

**3.** In the section 2 of results (line 285-line 296), 85 patients were identified infected by *Leishmania*, but in the section 3 (line 302-line313) and table 2 all the percentages were calculated based on the total 105 patients. In addition they have to add p value to show significant difference between sampling methods (line 307- line 308).

**Response:** On page 20, lines 386-389 we mentioned “When evaluating the discriminatory performance of the kDNA-PCR or the FRET probes-based Nested Real-Time PCR through the area under the ROC curve, no significant differences (p>0.050) were observed regardless of the sample type (Bx, FP, or L) or reference used (LCM or CRS).”

In Table 2 we could have added a third column considering only the 85 positive patients in kDNA-PCR and Nested RT-PCR, but as can be seen in the table attached below, the trend of greater positivity for lancet (L) with respect to filter paper (FP) and biopsy (Bx) is maintained if we compare with a total of 105 patients. As we mentioned above, at no time were the trials carried out with a priori knowledge of each other and all results remained blind to the operators at all times. We also confirmed if there was a misclassification of the 20 patients who tested negative, regardless of the denominator (105 or 85), with the same message: greater positivity in L > FP > Bx in both kDNA-PCR and Nested RT-PCR:

|  | **N=105** | **N=85** |
| --- | --- | --- |
| **Type of assay/analysis** | **n (%)** | **n (%)** |
| **Cell culture** | 41 (39.1) | 41 (48.2) |
| **kDNA-PCR** | | |
| Biopsy | 79 (75.2) | 79 (92.9) |
| Filter paper | 81 (77.1) | 81 (95.3) |
| Lancet | 84 (80.0) | 84 (98.8) |
| **Nested RT-PCR** | | |
| Biopsy | 58 (55.2) | 58 (68.2) |
| Filter paper | 57 (54.3) | 57 (67.1) |
| Lancet | 67 (63.8) | 67 (78.8) |
| **qPCR (parasite load)*** | | |
| Biopsy | 9.6 (7.3) |  |
| Filter paper | 10.0 (5.7) |  |
| Lancet | 9.9 (6.5) |  |
| **CRS** | | |
| CRS1 | 85 (81.0) |  |
| CRS2 | 85 (81.0) |  |
| *Log(*Leishmania* parasites per 10^6^ human cells) | | |

**4.** **Table 2** the parasite load is not clear, in my opinion they have to illustrate the parasite load separately in another figure.

**Response:** We consider that Figure 4 describes the parasite load according to sample type in pairs (4A), individually (4B), as well as stratified according to the intensity of the electrophoresis band (4C), page 18.

**5.** For the weak band intensity, it could be interesting to add another molecular method for the identification of parasite.

**Response:** Different methods have been developed for the enrichment of pathogenic DNA samples to sequence the genome of those samples with low parasitemia. We could apply these methodologies, but this will mean that the tests for the determination of *Leishmania* species are longer and more expensive. As we mentioned before, we were exploring high-throughput sequencing using different markers, but the intensity of the weak band has not yet been overcome.

**Minor point**

**1.** In my opinion the title is general, the authors should think about another title reflecting the message of the study

**Response:** Thank you for this suggestion, but we find that the title given is very useful and informative of what we have found and want to communicate.
