# Supplementary material for: Sampling is decisive to determination of Leishmania (Viannia) species
Source: PLoS Negl Trop Dis. 2024 Apr 25;18(4):e0012113. doi: 10.1371/journal.pntd.0012113 (PMC11045131; doi:10.1371/journal.pntd.0012113)
Supplement: Attachment — Submitted filename: Cover and responsesMar2024.docx [file pntd.0012113.s004.docx]

March 11th, 2024

(PNTD-D-23-01361) - [EMID:b2c56bb03b10b66c] manuscript "Sampling is decisive to determination of *Leishmania* species" for consideration at PLOS Neglected Tropical Diseases

Dear PLOS NTD Editors and Reviewers

We have made last appropriate modifications to the manuscript and are confident that we have answered all of suggestions of the reviewer #2.

Below we mention the responses and some modifications to the text.

Best regards

Maxy B. De los Santos D.

Principal and corresponding author

//

**Summary and General Comments**

**Reviewer #2:** The authors answered to all queries and included all the modifications requested. They improved the quality of the manuscript. Two points still need to be revised.

**Minor revision**

1. The title is still general, not limited to what was carried out. Indeed the authors used just Leishmania viannia species causing CL in Peru. I suggest ex “sampling is decisive to Leishmania viannia species identification…

**Answer:** the title was modified as “Sampling is decisive to determination of *Leishmania (Viannia)* species” in lines 1 and 2 page 1.

2. The authors answered that they used samples from healthy individuals as a negative controls. Please mention that in the text in MM (study population).

**Answer:** A paragraph was added in lines 133-134, page 7 "Additionally, blood samples were collected from healthy individuals from non-endemic area as negative control".
